# Evaluating Total Lymphocyte Count as a Surrogate Marker for CD4 Cell Count in the Management of HIV-Infected Patients in Resource-Limited Settings: A Study from China

**DOI:** 10.1371/journal.pone.0069704

**Published:** 2013-07-18

**Authors:** Jieqing Chen, Wei Li, Xiaojie Huang, Caiping Guo, Ran Zou, Qiuying Yang, Hongwei Zhang, Tong Zhang, Hui Chen, Hao Wu

**Affiliations:** 1 School of Biomedical Engineering, Capital Medical University, Beijing, China; 2 Department of Infectious Diseases, Beijing YouAn Hospital, Capital Medical University, Beijing, China; Alberta Provincial Laboratory for Public Health/University of Alberta, Canada

## Abstract

**Objective:**

To evaluate the correlation of total lymphocyte count (TLC) and CD4 cell count and the suitability of TLC as a surrogate marker for CD4 cell count of HIV-infected patients in China.

**Methods:**

Usefulness of TLC as a surrogate marker for a CD4 cell count <350 cells/mm^3^ for HIV-positive patients in China was evaluated by 977 pairs of TLC and CD4 cell count from 977 outpatients. The result was then validated by a literature review which was conducted on 9 relevant articles. Further investigation using the 977 pairs of TLC and CD4 cell count data was done to determine a TLC threshold for predicting a CD4 cell count <500 cells/mm^3^. Correlation and receiver operating characteristic (ROC) analysis were performed for both CD4 cell counts, and the sensitivity and specificity were computed.

**Results:**

Good correlation was noted between TLC and CD4 count (r = 0.60, 95% CI, 0.56–0.64). TLC obtained a relatively high diagnostic performance (area under ROC curve, 0.80) for predicting a CD4 cell count <350 cells/mm^3^, with a sensitivity of 0.65 (95% CI, 0.61–0.68) and a specificity of 0.80 (95% CI, 0.75–0.85) at the TLC threshold of 1570 cells/mm^3^. The literature review suggested that for a CD4 cell count <350 cells/mm^3^, the optimal TLC threshold was 1500 cells/mm^3^, which was similar to the figure presented in this observational study. As for predicting a CD4 cell count <500 cells/mm^3^, TLC obtained a high diagnostic performance (area under ROC curve, 0.82) as well with a sensitivity of 0.70 (95% CI, 0.67–0.73) and a specificity of 0.80 (95% CI, 0.73–0.87).

**Conclusions:**

When considering the antiretroviral therapy for HIV-infected Chinese individuals, total lymphocyte count can be considered as an inexpensive and easily available surrogate marker for predicting two clinically important thresholds of CD4 count of 350 cells/mm^3^ and 500 cells/mm^3^.

## Introduction

Globally, 34 million people were living with human immunodeficiency virus (HIV) at the end of 2011 [1]. Over 90% of HIV-infected people lived in low- and middle-income countries, and an estimated 14.2 million people in these countries required highly active antiretroviral therapy (HAART) [1]. Measures of CD4+ T-lymphocytes are used to guide clinical and therapeutic management of HIV-infected persons. Such measures are, however, frequently unavailable or too expensive for many regional hospitals or medical clinics in resource-limited settings [2], [3]. In April 2002, the World Health Organization (WHO) suggested that total lymphocyte count (TLC) could serve as a surrogate for CD4+ cell count [4] because TLC is easily obtained from routine complete blood cell counts by multiplying the percentage of lymphocytes by the white-blood-cell count. WHO recommended using a TLC of 1200 cells/mm^3^ as a surrogate marker for a CD4 count of 200 cells/mm^3^ for treatment initiation [5]. Several studies from different regions of the world have demonstrated a good correlation between TLC and CD4+ cell count [6], [7].

The 2008 recommendations of the International AIDS Society for the antiretroviral treatment of adult HIV infection [2] suggested that antiretroviral therapy be initiated before CD4 cell count declines to less than 350 cells/mm^3^. In patients with 350 CD4 cells/mm^3^ or more, the decision to begin therapy should be individualized based on the presence of comorbidities, risk factors for progression to AIDS and non-AIDS diseases, and patient readiness for treatment. The 2010 recommendations of the International AIDS Society [3] proposed therapy for asymptomatic patients with a CD4 cell count ≤500 cells/mm^3^, for all symptomatic patients, and for those with specific conditions and comorbidities. Further, therapy should also be considered for asymptomatic patients with a CD4 cell count >500 cells/mm^3^. To date, and to the best of our knowledge, while investigations from China and other countries and regions of the world have focused exclusively on determining a TLC equivalent for a CD4 cell count <200 cells/mm^3^ or <350 cells/mm^3^, no data on a TLC surrogate for CD4 cell count <500 cell/mm^3^ have been reported. In this paper, we first assessed the relationship between TLC and CD4 cell count and the effectiveness of TLC in identifying patients with a CD4 cell count of less than 350 cells/mm^3^ and 500 cells/mm^3^ respectively in China. We then systematically reviewed the literature on evaluating the usefulness of TLC as a surrogate marker for a CD4 cell count for HIV-positive patients in China to assess the agreement between our study’s results and those of other studies.

## Materials and Methods

### Study Population

Data for this study were collected from outpatients in the Infectious Disease Department, Beijing YouAn Hospital, Capital Medical University between 2005 and 2011. A total of 1059 treatment-naïve HIV-infected patients were included in the study for CD4+ cell count and absolute lymphocyte measurement. The study was approved by the Beijing YouAn Hospital Research Ethics Committee, and written informed consent was obtained from each subject. HIV seropositive individuals were diagnosed based on HIV antibody-Elisa tests and confirmed by Western Blot by the Beijing CDC. Inclusion criteria were at least 18 years of age and HIV-1 seropositivity. Exclusion criteria were pregnancy, history of antiretroviral therapy and opportunistic infections or malignancies at time of recruitment.

### Laboratory Methods

For each study subject, blood samples for CD4 cell count and TLC was collected on the same day in sterile vacuum tubes (containing K3 EDTA anti-freezing). TLC was measured by a 5-part diff (STK-S Beckman Coulter) automated blood-analyzer. The CD4+ cell count was done by FACScan (Becton-Dickinson, San Diego, CA) using ‘lyse-no-wash’ and tricolor monoclonal antibody techniques.

### Statistical Analysis

Correlation between CD4 cell count and TLC was evaluated using Spearman ranked correlation coefficient. Bi-normal ROC curve analysis was conducted to evaluate the diagnostic performance of TLC for predicting a CD4 cell count <350 cells/mm^3^ or <500 cells/mm^3^, respectively. The optimal TLC threshold was determined where the corresponding sensitivity was maximized while the corresponding specificity was not less than 0.80, meaning that the optimal TLC threshold had a predictive power of not less than 80% for CD4 cell counts <350 cells/mm^3^ or <500 cells/mm^3^.

Statistical analyses were performed using SPSS software (version 17.0, SPSS, Chicago, USA) and ROCKIT software (version 1.2B, Charles E. Metz, Department of Radiology, University of Chicago).

## Results

**Figure 1:**
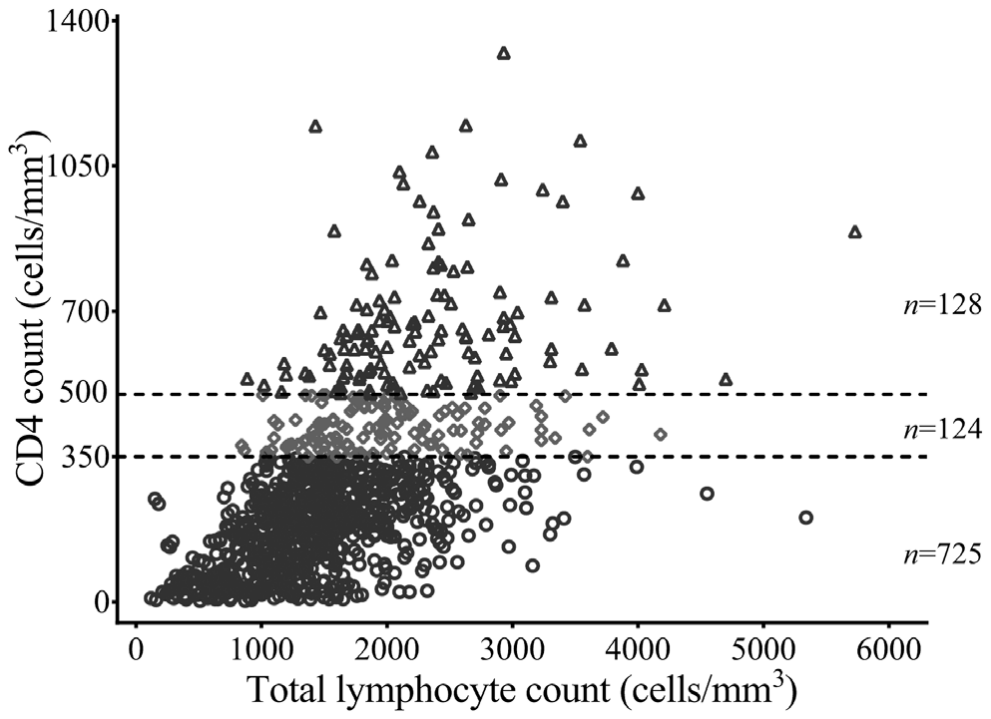
Scatter plot for 977 pairs of total lymphocyte count (TLC) and CD4 cell count. The lower circle dots are for 725 pairs of TLC (median [interquartile], 1370 [1025–1760] cells/mm^3^) and CD4 cell count of less than 350 cells/mm^3^. The middle diamond dots are for 124 pairs of TLC (median [interquartile], 1885 [1500–2355] cells/mm^3^) and CD4 cell count of between 350 cells/mm^3^ and 500 cells/mm^3^. The upper triangle dots are for 128 pairs of TLC (median [interquartile], 2222 [1840–2698] cells/mm^3^) and CD4 cell count of greater than 500 cells/mm^3^.

A total of 1059 observations were obtained from 1059 patients. Missing information for 21 CD4 cell counts and 61 paired white blood cell counts and TLCs resulted in 977 available paired TLCs and CD4 cell counts in this study, as shown in Figure 1. The baseline characteristics of these patients are listed in Table 1.

**Table 1:** Characteristics of the 977 HIV-infected patients included in the study.

Characteristic	Patients with CD4 count <350 cells/mm^3^	Patients with CD4 count ≥350 cells/mm^3^	Patients with CD4 count <500 cells/mm^3^	Patients with CD4 count ≥500 cells/mm^3^	Overall
Number (%)	725 (74.2%)	252 (25.8%)	849 (86.9%)	128 (13.1%)	977 (100%)
Age (year) †, median (IQR)	35 (28–42)	30 (25–37)	34 (28–41)	28 (24–37)	34 (27–41)
Male #, N (%)	662 (91.3%)	235 (93.3%)	772 (90.9%)	125 (97.7%)	897 (91.8%)
Co-infection					
Hepatitis B virus #, N (%)	67 (9.3%)	20 (7.9%)	78 (9.2%)	9 (6.8%)	87 (8.9%)
Hepatitis C virus #, N (%)	36 (5.0%)	7 (2.8%)	39 (4.6%)	4 (3.1%)	43 (4.4%)
Transmission #					
Transfusion, N (%)	42 (5.8%)	10 (4.0%)	45 (5.3%)	7 (5.5%)	52 (5.3%)
Drug injection, N (%)	22 (3.0%)	5 (2.0%)	24 (2.8%)	3 (2.3%)	27 (2.8%)
Homosexuality, N (%)	486 (67.0%)	192 (76.2%)	584 (68.8%)	94 (73.4%)	678 (69.4%)
Heterosexuality, N (%)	104 (14.3%)	28 (11.1%)	115 (13.5%)	17 (13.3%)	132 (13.5%)
Other, N (%)	71 (9.8%)	17 (6.7%)	81 (9.5%)	7 (5.5%)	88 (9.0%)
CD4 count (cells/mm^3^) †, median (IQR)	189 (88–263)	503 (414–639)	214 (107–305)	636 (552–732)	235 (130–358)
TLC (cells/mm^3^) †, median (IQR)	1370 (1025–1760)	2010 (1650–2590)	1460 (1100–1870)	2222 (1840–2698)	1520 (1140–1990)

† *P*<0.001 for difference between patients with and without CD4 count <350 cells/mm^3^ and patients with and without CD4 count <500 cells/mm^3^.

# *P*>0.05 for difference between patients with and without CD4 count <350 cells/mm^3^ and patients with and without CD4 count <500 cells/mm^3^.

IQR, interquartile range.

Spearman ranked correlation coefficients for all (977) pairs of TLC and CD4 cell count, for 849 (87.0%) pairs of TLC and CD4 cell count where the CD4 cell counts were less than 500 cells/mm^3^ and for 725 (74.2%) pairs of TLC and CD4 cell count where the CD4 cell counts were less than 350 cells/mm^3^ were 0.60 (95% CI, 0.56–0.64), 0.53 (95% CI, 0.48–0.58) and 0.49 (95% CI, 0.43–0.54), respectively. The correlation of TLC and CD4 at the CD4 threshold of 500 cells/mm^3^ was higher than that at the CD4 threshold of 350 cells/mm^3^ (P = 0.29), suggesting that using TLC for predicting a CD4 less than 500 cells/mm^3^ would be more reliable than using TLC for predicting a CD4 less than 350 cells/mm^3^.

**Figure 2:**
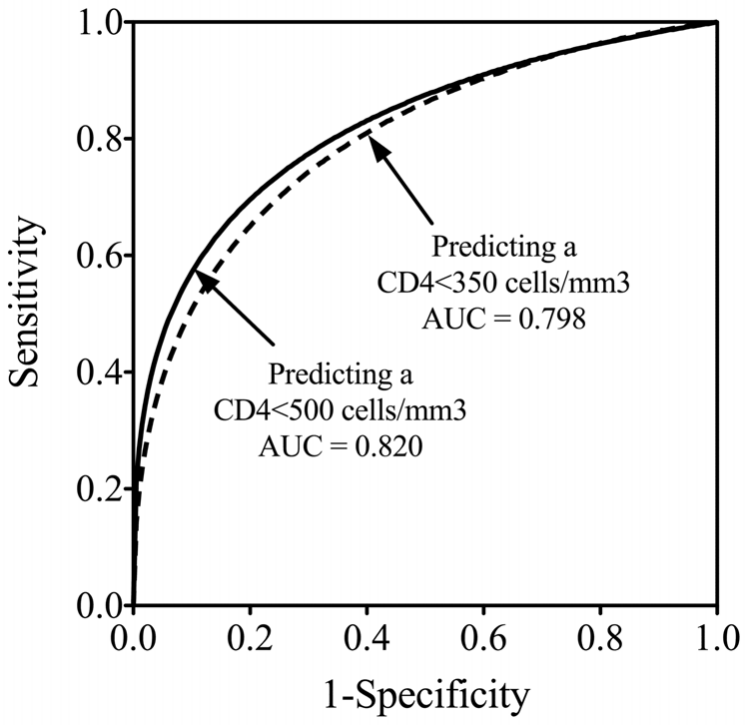
Receiver operating characteristic (ROC) curves for TLC thresholds predicting a CD4 cell count <350 cells/mm^3^ and a CD4 cell count <500 cells/mm^3^. Areas under ROC curves (AUCs) were 0.80 and 0.82, respectively.D.

The ROC curves for TLC predicting a CD4 cell count <350 cells/mm^3^ and a CD4 cell count <500 cells/mm^3^ are shown in Figure 2. The areas under ROC curves (AUC) were 0.80 (95% CI, 0.77–0.83) and 0.82 (95% CI, 0.78–0.85), respectively. The AUC values of 0.80 or 0.82 may be interpreted as the average sensitivity of 0.80 or 0.82 across all possible specificities and the average specificity of 0.80 or 0.82 across all possible sensitivities for TLC predicting a CD4 cell count <350 cells/mm^3^ or <500 cells/mm^3^, respectively.

Among the operating points derived from the ROC curves shown in Figure 2, it was found that using a TLC threshold of 1570 cells/mm^3^ would identify a patient who has a CD4 cell count <350 cells/mm^3^ with sensitivity 0.65 (95% CI, 0.61–0.68), specificity 0.80 (95% CI, 0.74–0.85), positive predictive value (PPV) 0.90 (95% CI, 0.87–0.93) and negative predictive value (NPV) 0.44 (95% CI, 0.40–0.49); using a TLC threshold of 1750 cells/mm^3^ would identify a patient who has a CD4 cell count <500 cells/mm^3^ with sensitivity 0.70 (95% CI, 0.67–0.73), specificity 0.80 (95% CI, 0.73–0.87), PPV 0.96 (95% CI, 0.94–0.97) and NPV 0.29 (95% CI, 0.24–0.34). Therefore, according to the criteria for the optimal TLC threshold mentioned in the Materials and Methods section (i.e. the optimal TLC threshold was determined where the corresponding sensitivity was maximized while the corresponding specificity was not less than 0.80), the optimal TLC thresholds for predicting a CD4 cell count less than 350 cells/mm^3^ and 500 cells/mm^3^ were determined at 1570 cells/mm^3^ and 1750 cells/mm^3^, respectively.

## Literature Review

Many investigations assessing the predictive power of TLC for a CD4 cell count less than 350 cells/mm^3^ from different areas of China have been reported with different numbers of subjects, giving different optimal TLC thresholds. Therefore, we systematically reviewed the relevant literature to estimate the usefulness of TLC as a surrogate marker for a CD4 cell count <350 cells/mm^3^ for Chinese HIV-infected patients.

### Literature Search and Study Selection

Literature searches covering articles published between 2000 and 2012 were performed in PubMed/MEDLINE, China National Knowledge Infrastructure (CNKI, http://www.cnki.net), and Wanfang Medicine Online (http://med.wanfangdata.com.cn), using the following key search words: ‘TLC or total lymphocyte count’ and ‘CD4 count’ and ‘HIV or AIDS’.

The titles and abstracts of 93 potentially relevant references were identified through the literature search and reviewed independently by two investigators in detail. Articles were excluded if: the study population was not Chinese HIV-infected persons (70 articles); sensitivity, specificity, positive predictive value (PPV) and negative predictive value (NPV) were not reported or could not be calculated from the information provided (2 articles); the relationship between the change of TLC and the change of CD4 cell count was investigated (6 articles); participants had started antiretroviral therapy (3 articles); or if CD4 cell counts were predicted by TLC combined with hemoglobin (3 articles). Thus, 9 primary articles [8]–[16] involving 2933 subjects with a total of 4855 pairs of TLC and CD4 cell count data were included in the analysis.

### Data Extraction

Study characteristics and accuracy data were collected from each selected article. Accuracy information, including sensitivity, specificity, PPV and NPV, were used to construct 2×2 tables of test results of different TLC thresholds (ranging from 1400 to 2200 cells/mm^3^) for predicting a CD4 cell count <350 cells/mm^3^ (testing positive if TLCs were above a threshold as defined in the primary study, and testing negative if these were below the threshold). Table 2 summarizes the basic features of each of the selected articles.

**Table 2:** Data from articles included in the literature review.

Author	Year	Area (Province)	No. ofsubjects	No. ofsamples	Samples (%) with CD4count <350 cells/mm^3^	TLC thresholds(cells/mm^3^)
Chang [8]	2007	Guangdong	602	1883	1508 (80.1)	1400 to 2200
Liu [9]	2004	Liaoning	75	167	96 (57.5)	1500 to 2200
Pan [10]	2009	Guangxi	136	144	77 (53.5)	1400 to 2200
Wu [11]	2010	Zhejiang	115	294	146 (49.7)	1700 to 2200
Xi [12]	2008	Beijing	778	1038	686 (66.1)	1400 to 1700
Xie [13]	2007	Beijing	317	317	221 (69.7)	1400 to 1900
Ye [14]	2006	Beijing	180	267	176 (65.9)	1500 to 2200
Zhang [15]	2008	Anhui	279	279	150 (53.8)	1500 to 2200
Zhou [16]	2007	Chongqing	451	466	255 (54.7)	1500 to 2200
Total			2933	4855	3315 (68.3)	

All statistical analyses were performed using Stata 12.0 statistical package.

### Results of Data Analysis

**Figure 3:**
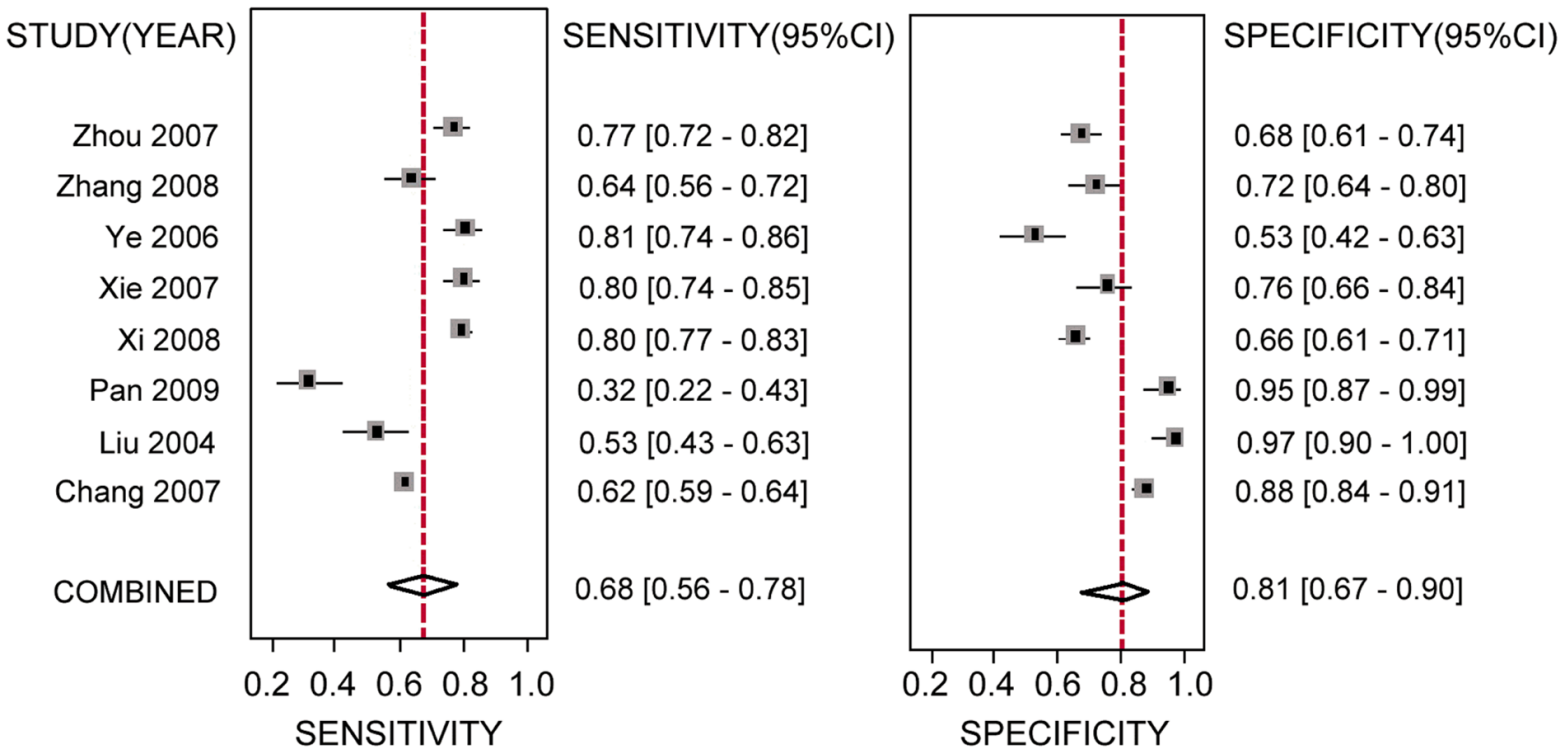
Forest plots of eight studies on a TLC threshold of 1500 cells/mm^3^ for predicting a CD4 cell count <350 cells/mm^3^. 95% confidence intervals (CIs) of sensitivity and specificity are in parentheses. The figure shows the estimated sensitivity and specificity of the study (square) with its 95% confidence interval (horizontal line). The pooled sensitivity and specificity were 0.68 (95 CI%, 0.56–0.78) and 0.81(95% CI, 0.67–0.90), respectively.

**Figure 4:**
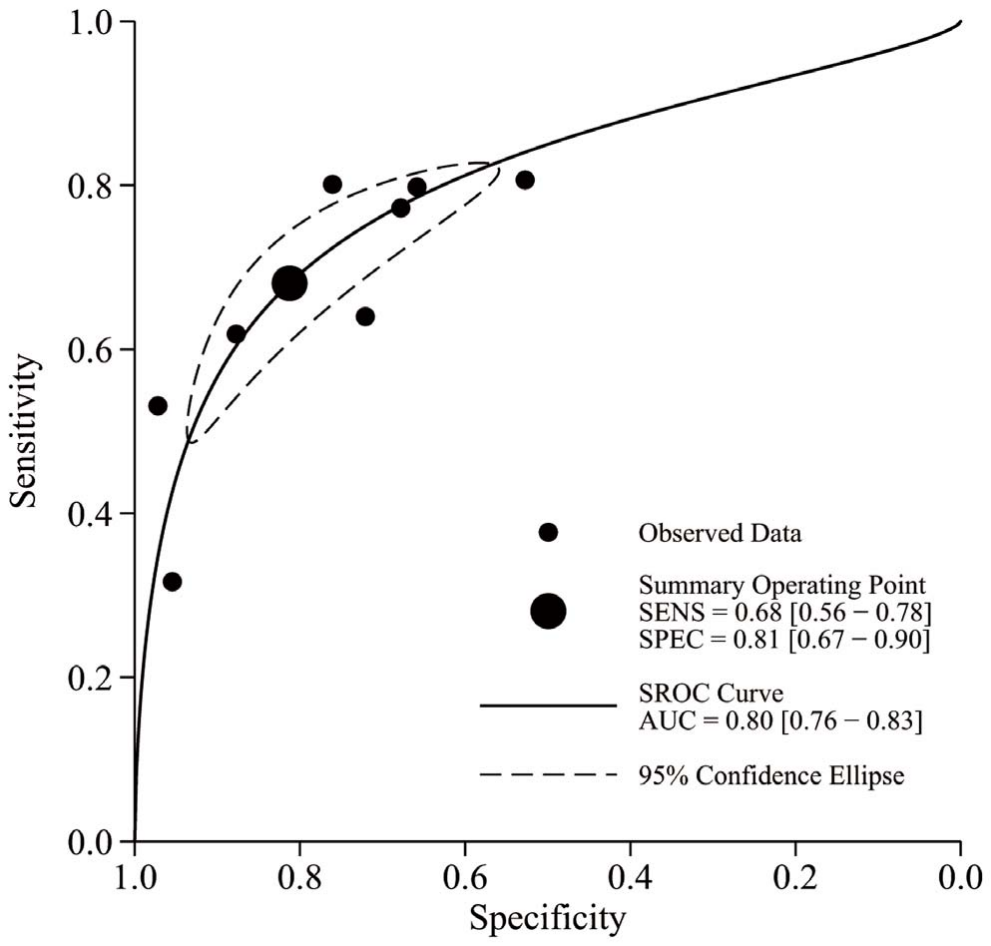
Summary receiver operating characteristic (SROC) plot of eight studies on a TLC threshold of 1500 cells/mm^3^ for predicting a CD4 cell count <350 cells/mm^3^. The area under the SROC curve (the solid line) is 0.80; the larger dot is the pooled value for sensitivity and specificity (0.68 and 0.81, respectively); the ellipse around the larger dot represents the 95% confidence interval around the summary estimates.

Sensitivity of the selected studies varied from 0.30 to 0.97 and specificity from 0.18 to 0.97 across the range of TLC thresholds. All of the pooled effect estimates when predicting a CD4 cell count <350 cells/mm^3^ by using different TLC thresholds are listed in Table 3. The pooled sensitivities and specificities and the areas under the summary ROC curves were shown as forest plots and the summary ROC curves. Figure 3 and Figure 4 are the forest plot and the summary ROC curve for the TLC threshold of 1500 cells/mm^3^ for predicting a CD4 cell count <350 cells/mm^3^, respectively. According to the criteria adopted in our study, the optimal TLC threshold was found to be 1500 cells/mm^3^ with a pooled sensitivity of 0.68 (95% CI, 0.56–0.78) and a pooled specificity of 0.81 (95% CI, 0.67–0.90), as shown in the second row in Table 3.

**Table 3:** Pooled effects for predicting a CD4 cell count <350 cells/mm^3^ by using different TLC thresholds.

TLC (cells/mm^3^)	No. of studies	Sensitivity (95% CI)	Specificity (95% CI)	Area under SROC curve (95% CI)
1400	4	0.60 (0.43–0.75)	0.89 (0.74–0.96)	0.81 (0.77–0.84)
**1500**	**8**	**0.68 (0.56–0.78)**	**0.81 (0.67–0.90)**	**0.80 (0.76–0.83)**
1600	7	0.76 (0.65–0.84)	0.70 (0.55–0.82)	0.80 (0.76–0.83)
1700	9	0.77 (0.67–0.84)	0.70 (0.56–0.80)	0.80 (0.76–0.83)
1800	8	0.80 (0.71–0.87)	0.65 (0.50–0.77)	0.80 (0.76–0.83)
1900	7	0.85 (0.75–0.91)	0.56 (0.39–0.71)	0.80 (0.76–0.83)
2000	6	0.85 (0.74–0.92)	0.50 (0.32–0.67)	0.76 (0.73–0.80)
2100	6	0.88 (0.79–0.94)	0.43 (0.28–0.60)	0.76 (0.72–0.80)
2200	7	0.91 (0.84–0.95)	0.41 (0.27–0.56)	0.79 (0.75–0.82)

SROC, summary receiver operating characteristic.

## Discussion

New HIV treatment guidelines highlighted that treatment should be started in patients with a CD4 cell count below 500 cells/mm^3^ [2]. Unfortunately, CD4 cell counts are unavailable or too expensive in many resource-limited settings. Evidence for the predictive worth of this TLC level was encouraging, with several large studies confirming the significant association between a TLC of <1200 cells/mm^3^ and subsequent disease progression or mortality [17], [18]. Others proposed that rate of TLC decline should be used in disease monitoring as a rapid decline (33% per year) precedes the onset of AIDS by 1–2 years [18]. Thus WHO suggested that TLC, which is more widely accessible and cheaper than CD4 cell counts, could serve as a potential marker of immunosuppression [5].

Many investigators from different countries and regions of the world have focused on evaluating the usefulness of TLC as a surrogate marker of a CD4 cell count <350 cells/mm^3^ for HIV infected patients of different ethnicities. Kumarasamy et al. [19] found that a TLC <1700 cells/mm^3^ had a 70% sensitivity, 86% specificity, 86% PPV and 69% NPV for predicting a CD4 cell count <350 cells/mm^3^ in India. Studies from Indonesia [20] and Brazil [21] also suggested that the 1700 cells/mm^3^ TLC was the optimal TLC threshold to identify patients with CD4 cell counts <350 cells/mm^3^, with a sensitivity of 81% and 59.4%, a specificity of 85% and 75.8%, a PPV of 96% and 57.3%, and an NPV of 51% and 79.4%, respectively. However, Moore et al. [22] found that a TLC threshold of 2250 cells/mm^3^ was the most accurate (73%) predictor of a CD4 cell count <350 cells/mm^3^, while yielding a sensitivity of 81% and a specificity of 54% in Uganda.

In this study, we evaluated the correlation between TLC and CD4 cell count and the effectiveness of TLC in identifying patients with a CD4 cell count <350 cells/mm^3^ by using data from outpatients in a hospital in Beijing, China. Sensitivity (65%) and specificity (80%) optimally aggregated at TLC <1570 cells/mm^3^ for CD4<350 cell/mm^3^. We further conducted a literature review on 9 studies evaluating the usefulness of TLC as a surrogate marker for a CD4 cell count <350 cells/mm^3^ for HIV-infected Chinese adults. According to the criteria used in our study, the optimal TLC threshold was determined at 1500 cells/mm^3^, giving a sensitivity of 68% and a specificity of 81%. Results of the optimal TLC thresholds and the corresponding combinations of sensitivity and specificity from the outpatient study and the results of the literature review were quite similar, while both were different from those reported among HIV-infected populations in other countries [19]–[22]. These differences are likely be due to the influence of certain patient characteristics on the relationship between TLC and CD4 cell count, the distribution of CD4 cell counts within a particular population and the male to female ratio of patients. The difference among the optimal TLC thresholds may also be a result of different criteria which were adopted while not stated explicitly in each study. However, the median (IQR) CD4 cell count of 796 (597–966) cells/mm^3^ in a group of 130 HIV-negative individuals in China was similar to those seen in western populations [23], which suggests that our observed difference may be not accounted for by background differences.

According to the latest recommendation of the International AIDS Society–USA Panel [3], therapy was recommended for asymptomatic patients with a CD4 cell count <500 cells/mm^3^. Our findings reveal that the utility of a TLC threshold of 1750 cells/mm^3^ for correlating with a CD4 cell count <500 cells/mm^3^ was adequate. Within the HIV-infected patients in this study, using a TLC threshold of 1750 cells/mm^3^ or less would identify the patient who has a CD4 cell count <500 cells/mm^3^ with sensitivity 70% and specificity 80%. The cutoff level is a balance between sensitivity and specificity in the relationship between TLC and CD4 cell count. No similar study on the use of TLC for predicting a CD4 cell count <500 cells/mm^3^ has previously been reported, to our best knowledge. This is the first study to highlight the usefulness of TLC as a surrogate marker for CD4 cell count for HIV-positive patients and it assessed the effectiveness of TLC in identifying patients with a CD4 cell count <500 cells/mm^3^ in China.

Conclusions regarding the usefulness of TLC for predicting CD4 cell count varied by study. Investigators whose results showed diverse combinations of sensitivity and specificity (70% and 86% [19] vs. 81% and 54% [22], for example) concluded that TLC was useful to predict eligibility for ART based on CD4 cell count criteria [19], [20], [22], [24]–[27]. Other studies drew the opposite conclusion, that TLC was not useful as an alternative to CD4 cell counts as a marker in HIV-infected patients [20], [28]–[30]. Chinese guidelines of treatment for AIDS proposed that antiretroviral therapy was recommended for asymptomatic patients with a CD4 cell count less than 350 cells/mm^3^, and antiretroviral therapy should be considered for asymptomatic patients with a CD4 cell count between 350 and 500 cells/mm^3^ [31]. Therefore, the findings in our study, that a TLC of 1570 cells/mm^3^ and 1750 cells/mm^3^ could predict a CD4 cell count <350 cells/mm^3^ and 500 cells/mm^3^ with the combined sensitivity and specificity of 65% and 80% and 70% and 80% respectively, could be very useful to the implementation of treatment in resource-limited areas without access to CD4 testing.
